# Sense of Belonging, Burnout, and Work Intentions Among US Physicians

**DOI:** 10.1001/jamanetworkopen.2026.4171

**Published:** 2026-03-30

**Authors:** Lindsey E. Carlasare, Purva Shah, Amanda Turzi, Nancy Nankivil, Jane Fogg, Lisa S. Rotenstein

**Affiliations:** 1 American Medical Association, Chicago, Illinois; 2 University of California San Francisco

## Abstract

**Importance:**

A sense of belonging is a fundamental need that predicts mental, physical, social, economic, and behavioral outcomes. Experiencing a sense of belonging at work may be influenced by organizational culture and whether a person feels supported by teammates. There is little evidence of what proportion of physicians feel a strong sense of belonging and teammate support. There exists a gap in literature showing whether these factors are associated with burnout or work intentions, which leaves health care leaders without a valuable tool to improve professional well-being and workforce retention.

**Objective:**

To provide health care leaders with a better understanding of how fostering belonging and teammate support can decrease burnout and potentially prevent costly turnover.

**Design, Setting, and Participants:**

This cross-sectional study used data from a survey administered to physicians by their organization leaders as part of the organization’s quality improvement efforts through participation in the American Medical Association Organizational Biopsy program. The survey was administered within health care organizations and health systems across the US with more than 50 physicians. The study population comprised physicians working in US-based health care organizations and health systems. Survey responses were collected between November 7, 2023, and November 12, 2024. Analysis was performed from June to August 2025.

**Main Outcomes and Measures:**

Belonging and teammate support perceptions were measured by asking participants to indicate how much they agree with 2 statements. Burnout was assessed using the single-item Mini Z assessment, and work intentions were indicated by likeliness to reduce clinical hours in the next 12 months or leave practice within the next 2 years. Odds ratios (ORs) and 95% CIs were used.

**Results:**

Of 14 051 physicians, 7315 were male (52.1%), 1978 were Asian (14.1%), 406 were Black or African American (2.9%), 425 were Latinx or Hispanic (3.0%), and 8681 were White (61.8%). Additionally, 4733 (33.7%) had 20 or more posttraining years of clinical practice and 4475 (31.8%) practiced in primary care. A strong sense of belonging was endorsed by 8425 (60.0%) and 11 293 (80.4%) perceived teammate support. Females (odds ratio [OR], 0.91, 95% CI, 0.84-0.98) and physicians with more than 5 years of clinical practice (eg, 6-10 years: OR, 0.76; 95% CI, 0.67-0.85; ≥20 years: OR, 0.87; 95% CI, 0.78-0.96) had lower odds of having strong sense of belonging. A strong sense of belonging was associated with lower odds of burnout (OR, 0.22; 95% CI, 0.21-0.24), lower odds of intent to reduce clinical hours (OR, 0.48; 95% CI, 0.44-0.53), and lower odds of intent to leave the organization (OR, 0.23; 95% CI, 0.21-0.26). Similar patterns were observed for factors associated with teammate support and for the association of teammate support with burnout, intent to reduce hours, and intent to leave the organization.

**Conclusions and Relevance:**

In this cross-sectional study of physicians in the US, a strong sense of belonging was associated with lower odds of burnout, intent to reduce clinical hours, and intent to leave the organization. These findings suggest that fostering belonging and teammate support can benefit physicians, care teams, patients, and organizations by potentially avoiding physician turnover. Organizations should prioritize belonging and teammate support in their efforts to improve culture, work environment, and physician well-being.

## Key Points

**Question:**

Are sense of belonging and teammate support associated with burnout and intent to leave or reduce hours among physicians?

**Findings:**

In this cross-sectional study of 14 051 physicians across 85 health care organizations, 60% of physicians experienced a strong sense of belonging and 80% felt their teammates have their back. Endorsing a sense of belonging and teammate support are associated with lower odds of burnout, intent to reduce hours, and intent to leave.

**Meaning:**

These findings suggest that as organizations strive to retain the physician workforce, leaders should prioritize belonging and teammate support in efforts to improve culture, work environment, and physician well-being.

## Teaser

This cross-sectional study examines the association of belonging and teammate support with burnout and turnover among US physicians.

## Introduction

Humans have an inherent need to belong. A sense of belonging is a subjective experience that reflects a person’s feeling of being accepted, connected, and important within the systems around them.^1^ It is fundamental for humans, and predicts numerous mental, physical, social, economic, and behavioral outcomes.^1^ People may experience belonging in many ways, including within family units, communities, and social and professional circles. It can also be found in groups sharing a common experience, such as among coworkers, where a sense of belonging can be influenced by organizational culture and whether people feel supported by their teammates.^2,3^ A sense of team support, which can influence a person’s sense of belonging at work, manifests from many factors, including the ability to rely on others, shared confidence that team members will perform their roles, and open and safe communication.^4^

Fostering belonging and a sense of team support within the workplace can improve professional satisfaction,^5,6^ increase productivity,^7^ reduce turnover,^8,9,10^ and lead to better mental,^11,12,13^ psychosocial,^12^ and physical health^11,14^ for workers. When a sense of belonging is absent, people may feel disconnected, lonely, or socially isolated, resulting in lower commitment to the organization,^15^ lower performance,^15,16^ higher risk of mental and physical health problems,^17,18^ and lower professional satisfaction.^16^ Despite evidence about the potential importance of a sense of belonging and team support in health care organizations,^4,19^ there is little evidence of what proportion of physicians feel a sense of belonging or supported by their teammates within their organization.

It is also unclear which physician characteristics may be associated with a greater vs lesser sense of belonging and team support, and how belonging perceptions are associated with burnout, intent to reduce hours (ITR), or intent to leave an organization (ITL) among physicians. This understanding is critical given (1) a known substantial prevalence of burnout^20^ and its association with intent to reduce hours and intent to leave the job^21,22^; (2) known differences in physician experiences across race,^23,24^ sex,^8,25,26,27,28,29,30,31^ career stage,^32^ and specialty^33,34,35,36^; and (3) a desire of organizational leaders to take concrete action in order to improve professional well-being and workforce retention. This study aims to provide health care leaders with a better understanding of how a sense of belonging and teammate support are associated with burnout and intent to leave or reduce clinical hours, and thus how focusing on these areas can ultimately contribute to improving physician experiences.

## Methods

### Survey Instrument and Participants

The American Medical Association (AMA) Organizational Biopsy is a free assessment tool used to support organizations in measuring and taking action to improve the well-being of their workforce.^37,38,39,40^ At the time the data for this analysis was collected, the survey was available from the AMA upon request to organizations with at least 50 physicians and/or advanced practice clinicians. The survey has 40 core items, including the Mini Z burnout assessment,^41^ a brief, validated instrument used to measure clinician satisfaction and burnout, and additional items about individual and organizational demographics, organizational culture, and professional characteristics. Each participating organization selects from a bank of questions to create a custom survey, then distributes the online survey to some or all of its physicians and advanced practice providers as part of its quality improvement efforts.

The present analysis was limited to data from physicians who responded to the survey between November 7, 2023, and November 12, 2024. Given the retrospective nature of the analysis, there was no predetermined sample size. This cross-sectional study followed the Strengthening the Reporting of Observational Studies in Epidemiology (STROBE) reporting guideline. The study was deemed to be not human participant research by the University of Illinois Chicago institutional review board. Participant consent was implied by completion of the survey.

#### Demographic and Professional Questions

Participants were asked in the survey to self-identify their sex (male, female, prefer not to answer, nonbinary, or genderqueer), race and ethnicity (American Indian or Alaska Native; Asian; Black or African American; Latinx, Latino, Latina, or Hispanic; Middle Eastern or North African; Native Hawaiian or Pacific Islander; White; more than 1 race or ethnicity; or some other race). Participants were also asked the number of years in posttraining clinical practice and specialty, which was subcategorized into primary care specialties, hospital-based, medical specialties, surgical specialties, obstetrics and gynecology, and psychiatry (Centers for Medicare & Medicaid Services, 2021, Medicare Data on Provider Practice and Specialty Classification).^42^

#### Belonging Perceptions: Sense of Belonging and Teammate Support

To measure sense of belonging and perceptions of teammate support, the survey included questions that were created specifically for the AMA Organizational Biopsy assessment. Participants were asked to rate on a scale of 1 to 5 their level of agreement with the following statements: in our organization “we have a strong sense of belonging” and “I believe my teammates have my back.” Respondents selecting 4 (ie, agree) or 5 (ie, strongly agree) on these items were considered to have a strong sense of belonging and a belief that their teammates had their back. We hereafter refer to these concepts collectively as belonging perceptions.

#### Burnout

Burnout was assessed using the single-item question from the Mini Z assessment, included in the Organizational Biopsy survey, which asks participants to select one answer of the following options, using their own definition of burnout: (1) “I feel completely burned out. I am at a point where I may need to seek help”; (2) “the symptoms of burnout that I’m experiencing won’t go away. I think about work frustrations a lot”; (3) “I am beginning to burnout and have one or more symptoms of burnout, eg, emotional exhaustion”; (4) “I am under stress, and don’t always have as much energy as I did, but I don’t feel burned out”; and (5) “I enjoy my work. I have no symptoms of burnout.” For analysis, burnout was defined to include responses 1, 2, or 3.

#### Work Intentions

Work intentions were assessed by asking respondents to indicate how likely they were to reduce their work hours (ITR) within the next 12 months, and how likely they were to leave their organization (ITL) within the next 2 years. Response options for both items were “(1) definitely,” “(2) likely,” “(3) moderate,” “(4) slight,” and “(5) none.” The “(1) likely” and “(2) definitely” responses were considered to affirm intent.

### Statistical Analysis

Descriptive statistics, including frequency counts and percentages, were used to identify the demographic characteristics of the sample, including sex, race, years in practice, and specialty. The distribution of responses to the questions about sense of belonging and whether teammates have their back was analyzed for the overall sample and across sex, race or ethnicity, career length, and specialty groups. Burnout, ITR, and ITL were also analyzed for the overall sample and across sex, race or ethnicity, career length, and specialty groups.

Subsequently, 2 sets of multivariable logistic regression models were developed. In the first set of models, the aim was to identify demographic and professional characteristics associated with 2 perceptions: sense of belonging and belief that teammates have their back.

The second set of models explored the association of belonging and teammate support with 3 outcomes: burnout, ITR, and ITL within the next 2 years. All models were adjusted for sex, race or ethnicity, years in practice, and specialty. In sensitivity analyses, multivariable models assessed the association between sense of belonging and teammate support, constructed as ordinal variables rather than binary variables, with burnout, ITR, and ITL as binary outcomes.

Analyses were conducted in SAS version 9.4 (SAS Institute), with 2-sided α = .05 deemed significant. Analysis was performed from June to August 2025.

## Results

### Sample Demographics

The survey was completed by 14 051 physicians from 85 organizations between November 7, 2023, and November 12, 2024. This represented a median (IQR) organizational response rate of 43.0% (31.5%-53.5%). Of 14 051 physicians, 7315 were male (52.1%), 1978 were Asian (14.1%), 406 were Black or African American (2.9%), 425 were Latinx or Hispanic (3.0%), and 8681 were White (61.8%). A plurality (4733 [33.7%]) had 20 or more posttraining years of clinical practice and practiced in primary care (4475 [31.8%]) (Table 1).

**Table 1.** Participant Demographics

Characteristic	Participant, No. (%)^a^
Total	14 051
Sex	
Male	7315 (52.1)
Female	5896 (42.0)
Prefer not to answer	441 (3.1)
Nonbinary, genderqueer, transgender, more than 1 gender^b^	33 (0.2)
Missing	366 (2.6)
Race or ethnicity	
American Indian or Alaska Native or Native Hawaiian or Pacific Islander^b^	39 (0.3)
Asian	1978 (14.1)
Black or African American	406 (2.9)
Latinx/o/a or Hispanic	425 (3.0)
Middle Eastern or North African	263 (1.9)
White	8681 (61.8)
Prefer not to answer	1246 (8.9)
More than 1 race	422 (3.0)
Of some other race	118 (0.8)
Missing	473 (3.4)
Years in practice posttraining, y	
1-5	2485 (17.7)
6-10	2254 (16.0)
11-15	2104 (15.0)
16-20	1605 (11.4)
≥20	4733 (33.7)
Missing	879 (6.2)
Specialty	
Primary care	4475 (31.8)
Hospital based	3446 (24.5)
Medical specialty	2234 (15.9)
Surgery specialty	1806 (12.9)
Obstetrics and gynecology	771 (5.5)
Psychiatry	315 (2.2)
Missing	1004 (7.1)

^a^ Proportions exclude missing values.

^b^ Pooled due to small sample sizes.

### Prevalence of Belonging Perceptions, Burnout, and Work Intentions

In this study, 8425 physicians (60.0%) reported a strong sense of belonging and 11 293 (80.4%) believed their teammates have their back. Burnout was reported by 6027 physicians (42.9%). In addition, 2800 of 12 785 (21.9%) indicated an ITR and 2063 of 13 753 (14.7%) reported an ITL their organization within the next 2 years. The frequency of these factors across all demographic and professional groups is shown in Table 2.

**Table 2.** Frequency Distribution of Belonging Perceptions, Burnout, and Work Intentions Across Demographic Groups

Characteristic	Physicians, No	Physicians, No. (%)^a^				
	Total	Strong sense of belonging	Believe teammates have their back	Burnout	Intent to reduce clinical hours^b^	Intent to leave organization^b^
Total	14 051	8425 (60.0)	11 293 (80.4)	6027 (42.9)	2800 (21.9)	2063 (14.7)
Sex						
Male	7315	4460 (61.0)	5945 (81.3)	2816 (38.5)	1455 (21.8)	1115 (15.2)
Female	5896	3520 (59.7)	4717 (80.0)	2746 (46.6)	1156 (21.6)	752 (12.8)
Prefer not to answer	441	154 (34.9)	285 (64.6)	285 (64.6)	112 (28.4)	129 (29.3)
Nonbinary, genderqueer, transgender, more than 1 gender^c^	33	14 (42.4)	22 (66.7)	16 (48.5)	7 (24.1)	11 (34.4)
Missing	366	277 (75.7)	324 (88.5)	164 (44.8)	70 (21.1)	56 (15.3)
Race or ethnicity						
American Indian or Alaska Native or Native Hawaiian or Pacific Islander^c^	39	24 (61.5)	29 (74.4)	19 (48.7)	10 (30.3)	7 (18.9)
Asian	1978	1270 (64.2)	1564 (79.1)	695 (35.1)	356 (19.5)	240 (12.1)
Black or African American	406	253 (62.3)	318 (78.3)	149 (36.7)	78 (20.6)	57 (14.0)
Latinx/o/a or Hispanic	425	257 (60.5)	335 (78.8)	168 (39.5)	88 (23.3)	54 (12.7)
Middle Eastern or North African	263	162 (61.6)	197 (74.9)	87 (33.1)	44 (18.5)	40 (15.2)
White	8681	5263 (60.6)	7180 (82.7)	3756 (43.3)	1703 (21.7)	1234 (14.2)
Prefer not to answer	1246	516 (41.4)	850 (68.2)	710 (57.0)	333 (29.6)	285 (22.9)
More than 1 race	422	270 (64.0)	332 (78.7)	186 (44.1)	73 (19.4)	58 (13.7)
Of some other race	118	76 (64.4)	90 (76.3)	39 (33.1)	19 (17.4)	15 (12.7)
Missing	473	334 (70.6)	398 (84.1)	218 (46.1)	96 (21.9)	73 (15.4)
Years in practice posttraining, y						
1-5	2485	1578 (63.5)	2089 (84.1)	958 (38.6)	407 (18.3)	320 (12.9)
6-10	2254	1274 (56.5)	1817 (80.6)	1062 (47.1)	406 (20.0)	275 (12.2)
11-15	2104	1198 (56.9)	1663 (79.0)	1038 (49.3)	376 (19.7)	216 (10.3)
16-20	1605	954 (59.4)	1263 (78.7)	732 (45.6)	256 (17.6)	163 (10.2)
≥20	4733	2864 (60.5)	3753 (79.3)	1815 (38.3)	1135 (26.5)	918 (19.4)
Missing	870	557 (64.0)	708 (81.4)	422 (48.5)	220 (25.3)	171 (19.7)
Specialty						
Primary care	4475	2736 (61.1)	3627 (81.1)	1973 (44.1)	833 (21.0)	613 (13.7)
Hospital based	3446	2019 (58.6)	2771 (80.4)	1475 (42.8)	799 (25.0)	523 (15.2)
Medical specialty	2234	1364 (61.1)	1793 (80.3)	919 (41.1)	387 (18.6)	302 (13.5)
Surgery specialty	1806	982 (54.4)	1411 (78.1)	752 (41.6)	339 (20.6)	286 (15.8)
Obstetrics and gynecology	771	469 (60.8)	613 (79.5)	338 (43.8)	149 (22.5)	109 (14.1)
Psychiatry	315	202 (64.1)	263 (83.5)	96 (30.5)	59 (21.1)	47 (14.9)
Missing	1004	653 (65.0)	815 (81.2)	474 (47.2)	234 (25.1)	183 (18.2)

^a^ Proportions exclude missing values.

^b^ Intent to reduce clinical hours question was answered by 12 785 respondents. Intent to leave practice question was answered by 13 753 respondents.

^c^ Pooled due to small sample sizes.

### Factors Associated with Belonging Perceptions

Multivariable analysis demonstrated that compared with their male counterparts, female physicians had lower odds of having a strong sense of belonging (OR, 0.91; 95% CI, 0.84-0.98) or believing that their teammates had their back (OR, 0.89; 95% CI, 0.81-0.98). Compared with White physicians, almost all racial or ethnic groups had lower odds of believing their teammates had their back, ranging from an OR of 0.48 (95% CI, 0.41-0.57) for those that preferred not to answer to an OR of 0.75 (95% CI, 0.66-0.85) for Asian physicians. Physicians who preferred not to disclose their sex and race or ethnicity also had lower odds of a strong sense of belonging (sex: OR, 0.59; 95% CI, 0.47-0.75; race or ethnicity: OR, 0.55; 95% CI, 0.48-0.63) and believing teammates had their back (sex: OR, 0.73; 95% CI, 0.57-0.93; race or ethnicity: OR, 0.48; 95% CI, 0.41-0.57).

Additionally, practicing in a surgery specialty compared with primary care was associated with lower odds of a strong sense of belonging (OR, 0.76; 95% CI, 0.68-0.85) and believing teammates have their back (OR, 0.81; 95% CI, 0.70-0.93). Those with more than 5 years of posttraining practice had between 13% to 24% and 20% to 30% lower odds of having a strong sense of belonging (eg, ≥20 years: OR, 0.87; 95% CI, 0.78-0.96; 6-10 years: OR, 0.76; 95% CI, 0.67-0.85) and believing teammates had their back (eg, 6-10 years, OR, 0.80; 95% CI, 0.69-0.93; ≥20 years: OR, 0.70; 95% CI, 0.61-0.80), respectively. Table 3 contains odds ratios of responses by demographic and professional groups.

**Table 3.** Multivariable Models for Association of Demographic and Professional Characteristics with Belonging Perceptions

Characteristic	Strong sense of belonging		Believe teammates have their back	
	OR (95% CI)	*P* value	OR (95% CI)	*P* value
Sex				
Female	0.91 (0.84-0.98)	.01	0.89 (0.81-0.98)	.02
Male	1 [Reference]	NA	1 [Reference]	NA
Prefer not to answer	0.59 (0.47-0.75)	<.001	0.73 (0.57-0.93)	.01
Nonbinary, genderqueer, transgender, more than 1 gender^a^	0.44 (0.22-0.90)	.03	0.47 (0.22-0.99)	.053
Race or ethnicity				
American Indian or Alaska Native or Native Hawaiian or Pacific Islander^a^	1.15 (0.59-2.23)	.68	0.70 (0.33-1.48)	.35
Asian	1.16 (1.05-1.29)	.01	0.75 (0.66-0.85)	<.001
Black or African American	1.07 (0.87-1.31)	.55	0.72 (0.56-0.92)	.01
Latinx/o/a or Hispanic	0.98 (0.80-1.20)	.85	0.73 (0.57-0.93)	.01
Middle Eastern or North African	1.01 (0.78-1.30)	.97	0.59 (0.44-0.78)	<.001
White	1 [Reference]	NA	1 [Reference]	NA
Prefer not to answer	0.55 (0.48-0.63)	<.001	0.48 (0.41-0.57)	<.001
More than 1 race	1.20 (0.97-1.49)	.09	0.81 (0.63-1.04)	.10
Of some other race	1.17 (0.79-1.74)	.43	0.64 (0.41-1.00)	.052
Years in practice posttraining				
1-5	1 [Reference]	NA	1 [Reference]	NA
6-10	0.76 (0.67-0.85)	<.001	0.80 (0.69-0.93)	.004
11-15	0.77 (0.68-0.87)	<.001	0.74 (0.63-0.86)	<.001
16-20	0.85 (0.75-0.97)	.02	0.73 (0.62-0.85)	<.001
≥20	0.87 (0.78-0.96)	.01	0.70 (0.61-0.80)	<.001
Specialty				
Primary care	1 [Reference]	NA	1 [Reference]	NA
Hospital based	0.92 (0.84-1.01)	.08	0.96 (0.85-1.08)	.46
Medical specialty	0.99 (0.89-1.10)	.89	0.95 (0.83-1.08)	.43
Surgery specialty	0.76 (0.68-0.85)	<.001	0.81 (0.70-0.93)	.003
Obstetrics and gynecology	1.00 (0.85-1.17)	.99	0.91 (0.75-1.10)	.32
Psychiatry	1.12 (0.88-1.43)	.34	1.15 (0.85-1.57)	.37

Abbreviation: OR, odds ratio.

^a^ Pooled due to small sample sizes.

### Association of Belonging Perceptions with Burnout and Work Intentions

In adjusted models, having a strong sense of belonging was associated with lower odds of burnout (OR 0.22, 95% CI, 0.21-0.24), lower odds of ITR (OR 0.48, 95% CI, 0.44-0.53), and lower odds of ITL (OR 0.23, 95% CI, 0.21-0.26) (Table 4 and eTable 1 in Supplement 1). Likewise, believing teammates had their back was also associated with lower odds of burnout (OR 0.29, 95% CI, 0.27-0.32), ITR (OR 0.55, 95% CI, 0.49-0.61), and ITL (OR 0.31, 95% CI, 0.28-0.35) (Table 4 and eTable 1 in Supplement 1). Full models containing all ORs are shown in eTables 1 and 2 in Supplement 1.

**Table 4.** Adjusted Odds for Association of Belonging Perceptions with Burnout, Intent to Reduce Hours, and Intent to Leave^a^^,^^b^

Outcome	Burnout		Intent to reduce clinical hours		Intent to leave organization	
	OR (95% CI)	*P* value	OR (95% CI)	*P* value	OR (95% CI)	*P* value
Sense of belonging						
Have strong sense of belonging	0.22 (0.21-0.24)	<.001	0.48 (0.44-0.53)	<.001	0.23 (0.21-0.26)	<.001
Do not have strong sense of belonging	1 [Reference]	NA	1 [Reference]	NA	1 [Reference]	NA
Teammate support						
Believe teammates have their back	0.29 (0.27-0.32)	<.001	0.55 (0.49-0.61)	<.001	0.31 (0.28-0.35)	<.001
Do not believe teammates have their back	1 [Reference]	NA	1 [Reference]	NA	1 [Reference]	NA

Abbreviation: OR, odds ratio.

^a^ Models adjusted for sex, race or ethnicity, years in posttraining clinical practice, and specialty.

^b^ Each OR is from a separate multivariable, adjusted model evaluating the association between a belonging perception and each of the outcomes (burnout, intent to reduce clinical hours, or intent to leave organization).

### Sensitivity Analysis

In sensitivity analyses where a sense of belonging and teammate support were specified as ordinal rather than binary variables and associated with burnout, ITR, and ITL in multivariable logistic regression analyses, findings were consistent with the main model specifications described in the Results. Additional data can be found in eTable 3 and eTable 4 in Supplement 1.

## Discussion

In this multisite study of 14 051 physicians, high levels of a sense of belonging and perception of teammate support were observed. Specifically, nearly two-thirds of physicians surveyed reported experiencing a strong sense of belonging within their organizations and 4 of 5 felt their teammates have their back. Endorsing a sense of belonging and feeling one’s teammates have their back were associated with lower odds of burnout, ITR, and ITL. However, specific groups were less likely to endorse belonging perceptions, including female physicians, those in surgery specialties, those preferring not to disclose their sex or race and ethnicity, and physicians practicing for more than 5 years.

It is encouraging that high proportions of physicians surveyed experience a strong sense of belonging and feel their teammates have their back. Yet, there is still work to be done, as nearly 4 of 10 physicians do not feel a strong sense of belonging in their organization.

The differences in sense of belonging among certain groups, such as female physicians, may be driven by a variety of factors. While our study design and analysis did not allow for the determination of causality between outcomes and specific drivers, inference about potential contributing factors can be drawn from examples of systemic and cultural issues women commonly face in the health care workplace, as described in prior literature. For example, female physicians may feel minimized in certain male-dominated specialties^30^ and are more likely to experience disrespect and harassment at work.^27^ Female physicians are more likely than male physicians to work part-time,^26^ possibly preventing them from participating in work activities, having time to build relationships at work, or feeling like part of the community at work. Female physicians may be prevented from dedicating time to build strong connections at work more than males, as evidence shows that women physicians comparatively spend more time on family and home-related responsibilities.^28,43^ Despite having better patient outcomes,^44^ female physicians are more likely than male physicians to leave clinical practice,^45^ an outcome organizations could potentially prevent by enhancing opportunities for belonging, collegiality, and inclusion in the workplace.

Differences in belonging perceptions varied across specialties, particularly among physicians working in surgery specialties, who had lower odds of having a sense of belonging and feeling their teammates have their back. This observation may be attributed to certain professional or job-related factors. For example, surgical practice can require long hours^46^ and high physical and cognitive burden,^33^ and like many physicians, surgeons may experience overwork and burnout,^33,34^ potentially diminishing their sense of belonging within their organization or team.

It is notable that several racial and ethnic groups and those who prefer to not disclose their sex or race and ethnicity were less likely to perceive that teammates had their back. This is consistent with other literature demonstrating racial and ethnic minority physicians often feel unseen, excluded, and undervalued in the workplace.^19,47,48^ Future qualitative work may be helpful to reveal the specific experiences of these groups, as well as how they align with their demographic characteristics.

Career stage may also influence whether a physician feels a sense of belonging at work. Physicians with more than 5 years of posttraining clinical practice had lower odds of feeling belonging and teammate support than those with fewer than 5 years of practice. Coming out of residency, new physicians may still maintain relationships with peers and feel a heightened sense of enthusiasm and collegiality. New physicians may also receive a high degree of attention through onboarding and mentorship activities. Perhaps over time, increased workload, exposure to new practice demands, competition among colleagues, or isolation within their specialty reduce this sense of engagement with the workplace. Additionally, more physicians than ever are employed by large health systems,^49^ and modern health care is increasingly transactional, having shifted from a medical community to a fragmented system in which rates of loneliness and social isolation among physicians have increased.^50^ The current health care environment may drive more seasoned physicians to feel less engaged with their organization and colleagues.

This analysis reveals important associations between sense of belonging and burnout, ITR, and ITL, which have significant implications for the physician workforce. Our findings reflect those of previous smaller studies showing that workplace belonging is a significant factor of intent to leave the organization^8^ and that lower satisfaction with belonging is associated with greater intent to leave.^51^ Another study, limited to academic faculty physicians, demonstrated that greater peer support was associated with lower intent to leave.^21^ Other evidence shows burnout and ITL are strong estimators of actual departure from an organization,^35,52,53^ emphasizing the importance of our findings. Furthermore, the downstream effects of physician burnout and turnover can be seen in access-to-care issues^54,55,56^ and in excess costs incurred by health care organizations.^57,58^ These findings may provide insights into efforts that organizations can make to avoid costly physician turnover and increase retention rates by fostering a sense of belonging in the clinical workplace, promoting more opportunities for inclusion, and encouraging collegiality among clinicians, care team members, and other employees.

To nurture the organizational culture and create a supportive workplace for all health care workers that promotes belonging, organizational leadership should infuse the practice environment with people-focused policies and practices.^59^ Specifically, organizations can create, through transparent and thoughtful communication,^60^ a culture that promotes psychological safety, inclusion, and trust. Another beneficial tactic is to promote recognition^60^ and communicate the importance of individual contributions to the greater organizational purpose. Implementing a team-based culture where possible can foster strong collaboration in which physicians and team members can rely confidently on one another.^9^ Organizations should ensure physicians across demographics, career stages, and specialties are included and given opportunities to share experiences, provide feedback on organizational culture, and be involved in efforts to improve physician well-being.^10^ Since a sense of belonging within the organization may reflect a more global organizational culture, while feeling one’s teammates have their back may reflect a local team culture, improvements at the local level may also be justified. Providing opportunities for teamwork and cross-team interaction,^1,61,62^ such as team huddles that improve communication and efficiency,^63^ or team bonding and social events that promote collaboration, can generate cohesion and foster stronger relationships. Finally, organizational leadership should acknowledge the intersection of personal and professional identities and the negative and positive effects they can have on physicians’ experiences.^47^

### Strengths and Limitations

Strengths of this study include its large sample size, representing physicians and organizations across the US, as well as its high response rate. This study also has limitations. The sample is a convenience sample composed of physicians working at organizations that are intentionally measuring physician well-being as part of quality improvement efforts. The sample is not representative of all US physicians; thus, response bias is possible, and findings cannot be generalized to all US physicians.

## Conclusions

In this cross-sectional study of 14 051 physicians across 85 US health care organizations, 60% of physicians surveyed reported a strong sense of belonging within their organizations, and 80% felt their teammates had their back. Endorsing a sense of belonging and feeling that teammates have their back were associated with lower odds of burnout, ITR, and ITL. Specific physician groups were less likely to endorse belonging perceptions, including female physicians, those in surgery specialties, those preferring not to disclose their sex or race and ethnicity, and physicians practicing for more than 5 years. These findings have important implications for physician well-being and the physician workforce. Health care organizations should prioritize belonging and teammate support in their efforts to improve organizational culture, the work environment, and physician well-being. These factors will prove to be valuable as organizations strive to retain a diverse and healthy physician workforce.

## Notes

Supplement 1. **eTable 1. **Full Multivariable Logistic Regression Models Depicting Association Between a Strong Sense of Belonging and Burnout, ITR, and ITL, Adjusting for Demographic and Professional Factors **eTable 2. **Full Multivariable Logistic Regression Models Depicting Association Between Teammate Support and Burnout, ITR, and ITL, Adjusting for Demographic and Professional Factors **eTable 3. **Logistic Regression Models Depicting Association Between Sense of Belonging on an Ordinal Scale and Burnout, ITR, and ITL, Adjusting for Demographic and Professional Factors **eTable 4. **Logistic Regression Models Depicting Association Between Teammate Support on an Ordinal Scale and Burnout, ITR, and ITL, Adjusting for Demographic and Professional Factors

Supplement 2.
Data Sharing Statement
