# Supplementary material for: Sense of Belonging, Burnout, and Work Intentions Among US Physicians
Source: JAMA Netw Open. 2026 Mar 30;9(3):e264171. doi: 10.1001/jamanetworkopen.2026.4171 (PMC13036579; doi:10.1001/jamanetworkopen.2026.4171)
Supplement: Supplement 1. — eTable 1. Full Multivariable Logistic Regression Models Depicting Association Between a Strong Sense of Belonging and Burnout, ITR, and ITL, Adjusting for Demographic and Professional Factors eTable 2. Full Multivariable Logistic Regression Models Depicting Association Between Teammate Support and Burnout, ITR, and ITL, Adjusting for Demographic and Professional Factors eTable 3. Logistic Regression Models Depicting Association Between Sense of Belonging on an Ordinal Scale and Burnout, ITR, and ITL, Adjusting for Demographic and Professional Factors eTable 4. Logistic Regression Models Depicting Association Between Teammate Support on an Ordinal Scale and Burnout, ITR, and ITL, Adjusting for Demographic and Professional Factors [file jamanetwopen-e264171-s001.pdf]

Supplemental Online Content

Carlasare LE, Shah P, Turzi A, Nankivil N, Fogg J, Rotenstein L. Sense of belonging, burnout and work intentions among US physicians. *JAMA Netw Open*. 2026;9(3):e264171. doi:10.1001/jamanetworkopen.2026.4171

eTable 1. Full Multivariable Logistic Regression Models Depicting Association Between a Strong Sense of Belonging and Burnout, ITR, and ITL, Adjusting for Demographic and Professional Factors

eTable 2. Full Multivariable Logistic Regression Models Depicting Association Between Teammate Support and Burnout, ITR, and ITL, Adjusting for Demographic and Professional Factors

eTable 3. Logistic Regression Models Depicting Association Between Sense of Belonging on an Ordinal Scale and Burnout, ITR, and ITL, Adjusting for Demographic and Professional Factors

eTable 4. Logistic Regression Models Depicting Association Between Teammate Support on an Ordinal Scale and Burnout, ITR, and ITL, Adjusting for Demographic and Professional Factors

This supplemental material has been provided by the authors to give readers additional information about their work.

© 2026 Carlasare LE et al. *JAMA Network Open*.

eTable 1. Full Multivariable Logistic Regression Models Depicting Association Between A Strong Sense of Belonging and Burnout, ITR, and ITL, Adjusting for Demographic and Professional Factors

	**Burnout**		**Intent to Reduce Clinical Hours**		**Intent to Leave Organization**	
	OR (95% CI)	**p-Value**	OR (95% CI)	**p-Value**	OR (95% CI)	**p-Value**
**Sense of Belonging**						
Have strong sense of belonging (vs. Do not have strong sense of belonging)	0.22 (0.21-0.24)	<.001	0.48 (0.44-0.53)	<.001	0.23 (0.21-0.26)	<.001
**Sex (vs. Male)**						
Female	1.39 (1.28-1.51)	<.001	1.06 (0.96-1.16)	0.28	0.88 (0.79-0.99)	0.03
Prefer not to answer	1.76 (1.36-2.27)	<.001	0.93 (0.71-1.23)	0.62	1.59 (1.20-2.10)	0.001
Non-binary/Genderqueer/Transgender/> 1 gender†	1.03 (0.49-2.15)	0.95	0.93 (0.37-2.36)	0.89	2.35 (1.05-5.24)	0.04
**Race/Ethnicity (vs. White)**						
American Indian or Alaska Native/Native Hawaiian or Pacific Islander†	1.23 (0.63-2.43)	0.55	1.51 (0.69-3.33)	0.3	1.50 (0.62-3.63)	0.37
Asian	0.67 (0.59-0.75)	<.001	0.94 (0.82-1.07)	0.34	0.99 (0.84-1.16)	0.90
Black or African American	0.70 (0.56-0.88)	0.002	0.91 (0.70-1.19)	0.49	1.09 (0.80-1.48)	0.57
Latinx/Latino/Latina_Hispanic	0.83 (0.67-1.04)	0.10	1.12 (0.87-1.44)	0.39	0.94 (0.69-1.28)	0.68
Middle Eastern or North African	0.64 (0.48-0.85)	0.002	0.87 (0.62-1.23)	0.44	1.23 (0.85-1.77)	0.28
Prefer not to answer	1.20 (1.03-1.40)	0.02	1.43 (1.20-1.69)	<.001	1.33 (1.09-1.61)	0.004
More than 1 race	1.01 (0.81-1.26)	0.91	0.91 (0.69-1.20)	0.5	0.97 (0.70-1.34)	0.84
Of some other race	0.65 (0.43-1.01)	0.05	0.82 (0.49-1.38)	0.45	1.05 (0.59-1.86)	0.88
**Years in Practice Post Training (vs. 1-5 years)**						
6-10 years	1.33 (1.17-1.50)	<.001	1.05 (0.90-1.23)	0.55	0.84 (0.70-1.01)	0.06
11-15 years	1.46 (1.28-1.66)	<.001	1.03 (0.88-1.21)	0.68	0.69 (0.57-0.83)	<.001
16-20 years	1.30 (1.13-1.49)	0.002	0.91 (0.76-1.08)	0.29	0.69 (0.56-0.85)	0.001
20+ years	0.95 (0.85-1.06)	0.32	1.62 (1.42-1.85)	<.001	1.64 (1.42-1.91)	<.001
**Specialty (vs. Primary Care)**						
Hospital Based	0.91 (0.82-1.01)	0.07	1.30 (1.16-1.46)	<.001	1.15 (1.01-1.32)	0.04
Medical Specialty	0.91 (0.81-1.02)	0.10	0.89 (0.77-1.02)	0.09	1.01 (0.86-1.19)	0.88
Surgery Specialty	0.83 (0.74-0.94)	0.004	0.95 (0.82-1.11)	0.53	1.11 (0.94-1.30)	0.23
Obstetrics And Gynecology	0.93 (0.79-1.10)	0.39	1.10 (0.90-1.35)	0.36	1.12 (0.89-1.41)	0.35
Psychiatry	0.56 (0.43-0.73)	<.001	1.07 (0.79-1.45)	0.66	1.18 (0.84-1.66)	0.34

† Pooled due to small sample sizes.

© 2026 Carlasare LE et al. *JAMA Network Open*.

eTable 2. Full Multivariable Logistic Regression Models Depicting Association Between Teammate Support and Burnout, ITR, and ITL, Adjusting for Demographic and Professional Factors

	Burnout		Intent to Reduce Clinical Hours		Intent to Leave Organization	
	OR (95% CI)	**p-Value**	OR (95% CI)	**p-Value**	OR (95% CI)	**p-Value**
**Teammate Support**						
Believe teammates have their back (vs. Do not believe teammates have their back)	0.29 (0.27-0.32)	<.001	0.55 (0.49-0.61)	<.001	0.31 (0.28-0.35)	<.001
**Sex (vs. Male)**						
Female	1.38 (1.27-1.49)	<.001	1.06 (0.97-1.17)	0.22	0.90 (0.80-1.00)	0.06
Prefer not to answer	1.90 (1.49-2.44)	<.001	0.97 (0.74-1.28)	0.83	1.69 (1.28-2.24)	<.001
Non-binary/Genderqueer/Transgender/> 1 gender†	1.14 (0.55-2.37)	0.72	0.95 (0.38-2.39)	0.91	2.53 (1.14-5.62)	0.02
**Race/Ethnicity (vs. White)**						
American Indian or Alaska Native/Native Hawaiian or Pacific Islander†	1.08 (0.55-2.10)	0.82	1.41 (0.64-3.08)	0.39	1.35 (0.57-3.19)	0.50
Asian	0.61 (0.55-0.68)	<.001	0.89 (0.78-1.02)	0.09	0.88 (0.75-1.04)	0.13
Black or African American	0.66 (0.53-0.82)	<.001	0.87 (0.67-1.14)	0.32	0.99 (0.73-1.34)	0.95
Latinx/Latino/Latina_Hispanic	0.80 (0.64-0.98)	0.03	1.08 (0.84-1.40)	0.54	0.87 (0.64-1.19)	0.38
Middle Eastern or North African	0.58 (0.44-0.77)	<.001	0.84 (0.59-1.18)	0.31	1.11 (0.77-1.59)	0.58
Prefer not to answer	1.27 (1.09-1.47)	0.002	1.47 (1.24-1.74)	<.001	1.37 (1.13-1.66)	0.001
More than 1 race	0.91 (0.73-1.12)	0.37	0.86 (0.65-1.14)	0.28	0.87 (0.63-1.20)	0.39
Of some other race	0.58 (0.38-0.88)	0.01	0.77 (0.46-1.29)	0.32	0.90 (0.51-1.59)	0.72
**Years in Practice Post Training (vs. 1-5 years)**						
6-10 years	1.38 (1.22-1.56)	<.001	1.08 (0.92-1.26)	0.35	0.89 (0.74-1.06)	0.20
11-15 years	1.47 (1.30-1.66)	<.001	1.04 (0.89-1.22)	0.59	0.71 (0.59-0.85)	<.001
16-20 years	1.27 (1.11-1.45)	0.001	0.90 (0.76-1.08)	0.26	0.68 (0.55-0.83)	<.001
20+ years	0.93 (0.84-1.04)	0.20	1.59 (1.4-1.82)	<.001	1.57 (1.35-1.81)	<.001
**Specialty (vs. Primary Care)**						
Hospital Based	0.94 (0.85-1.03)	0.19	1.32 (1.17-1.48)	<.001	1.18 (1.03-1.35)	0.02
Medical Specialty	0.91 (0.81-1.01)	0.08	0.89 (0.77-1.02)	0.09	1.00 (0.86-1.17)	0.98
Surgery Specialty	0.90 (0.80-1.01)	0.07	0.98 (0.85-1.14)	0.83	1.17 (0.99-1.37)	0.06
Obstetrics And Gynecology	0.92 (0.78-1.08)	0.29	1.09 (0.89-1.33)	0.40	1.09 (0.87-1.37)	0.44
Psychiatry	0.57 (0.44-0.74)	<.001	1.07 (0.79-1.44)	0.68	1.18 (0.85-1.64)	0.33

† Pooled due to small sample sizes.

© 2026 Carlasare LE et al. *JAMA Network Open*.

eTable 3. Logistic Regression Models Depicting Association Between Sense of Belonging on an Ordinal Scale and Burnout, ITR, and ITL, Adjusting for Demographic and Professional Factors

	Burnout		Intent to Reduce Clinical Hours		Intent to Leave Organization	
	OR (95% CI)	p-Value	OR (95% CI)	p-Value	OR (95% CI)	p-Value
**Sense of Belonging**						
Have strong sense of belonging (vs. Do not have strong sense of belonging)	0.42 (0.41-0.43)	<.001	0.69 (0.67-0.71)	<.001	0.41 (0.40-0.43)	<.001
**Sex (vs. Male)**						
Female	1.43 (1.33-1.53)	<.001	1.07 (1.00-1.15)	0.07	0.94 (0.88-1.01)	0.08
Prefer not to answer	1.68 (1.36-2.07)	<.001	1.00 (0.80-1.24)	0.99	1.12 (0.91-1.39)	0.29
Non-binary/Genderqueer/Transgender/> 1 gender†	1.41 (0.75-2.68)	0.29	1.07 (0.54-2.12)	0.85	1.37 (0.71-2.61)	0.35
**Race/Ethnicity (vs. White)**						
American Indian or Alaska Native/Native Hawaiian or Pacific Islander†	0.84 (0.46-1.51)	0.55	1.36 (0.73-2.55)	0.34	0.91 (0.49-1.69)	0.76
Asian	0.63 (0.57-0.69)	<.001	0.91 (0.82-1.00)	0.05	0.86 (0.78-0.95)	0.003
Black or African American	0.68 (0.56-0.82)	<.001	0.85 (0.70-1.04)	0.11	0.83 (0.68-1.00)	0.05
Latinx/Latino/Latina_Hispanic	0.70 (0.58-0.85)	<.001	1.04 (0.86-1.26)	0.69	1.06 (0.88-1.28)	0.54
Middle Eastern or North African	0.62 (0.49-0.78)	<.001	0.90 (0.70-1.15)	0.39	0.96 (0.75-1.22)	0.72
Prefer not to answer	1.10 (0.97-1.26)	0.14	1.32 (1.15-1.51)	<.001	1.29 (1.13-1.47)	<.001
More than 1 race	1.03 (0.85-1.24)	0.79	0.92 (0.75-1.12)	0.40	1.03 (0.85-1.24)	0.80
Of some other race	0.52 (0.37-0.74)	<.001	0.87 (0.60-1.25)	0.44	0.79 (0.55-1.13)	0.20
**Years in Practice Post Training (vs. 1-5 years)**						
6-10 years	1.31 (1.18-1.46)	<.001	1.11 (0.99-1.24)	0.07	0.93 (0.84-1.04)	0.20
11-15 years	1.35 (1.21-1.51)	<.001	1.05 (0.94-1.18)	0.38	0.84 (0.75-0.94)	0.002
16-20 years	1.25 (1.11-1.41)	<.001	0.92 (0.81-1.04)	0.19	0.78 (0.69-0.88)	<.001
20+ years	0.86 (0.78-0.94)	0.001	1.55 (1.40-1.71)	<.001	1.33 (1.21-1.46)	<.001
**Specialty (vs. Primary Care)**						
Hospital Based	0.90 (0.83-0.98)	0.01	1.16 (1.07-1.27)	<.001	1.20 (1.10-1.31)	<.001
Medical Specialty	0.88 (0.80-0.97)	0.01	0.92 (0.83-1.01)	0.09	1.05 (0.96-1.16)	0.30
Surgery Specialty	0.86 (0.77-0.95)	0.004	0.85 (0.76-0.95)	0.003	1.20 (1.08-1.34)	0.001
Obstetrics And Gynecology	0.93 (0.81-1.07)	0.33	0.91 (0.78-1.07)	0.25	1.00 (0.86-1.15)	0.95
Psychiatry	0.64 (0.51-0.79)	<.001	0.96 (0.77-1.20)	0.72	1.38 (1.12-1.72)	0.003

† Pooled due to small sample sizes.

© 2026 Carlasare LE et al. *JAMA Network Open*.

eTable 4. Logistic Regression Models Depicting Association Between Teammate Support on an Ordinal Scale and Burnout, ITR, and ITL, Adjusting for Demographic and Professional Factors

	Burnout		Intent to Reduce Clinical Hours		Intent to Leave Organization	
	OR (95% CI)	p-Value	OR (95% CI)	p-Value	OR (95% CI)	p-Value
**Teammate Support**						
Believe teammates have their back (vs. Do not believe teammates have their back)	0.48 (0.46-0.50)	<.001	0.70 (0.68-0.73)	<.001	0.50 (0.48-0.52)	<.001
**Sex (vs. Male)**						
Female	1.40 (1.31-1.50)	<.001	1.07 (0.99-1.15)	0.07	0.94 (0.88-1.01)	0.10
Prefer not to answer	1.90 (1.54-2.34)	<.001	1.06 (0.86-1.32)	0.58	1.32 (1.07-1.63)	0.01
Non-binary/Genderqueer/Transgender/> 1 gender†	1.52 (0.80-2.86)	0.20	1.06 (0.54-2.08)	0.88	1.55 (0.82-2.93)	0.18
**Race/Ethnicity (vs. White)**						
American Indian or Alaska Native/Native Hawaiian or Pacific Islander†	0.84 (0.47-1.50)	0.55	1.32 (0.71-2.47)	0.38	0.93 (0.51-1.70)	0.80
Asian	0.57 (0.52-0.63)	<.001	0.86 (0.78-0.95)	0.003	0.77 (0.70-0.84)	<.001
Black or African American	0.60 (0.50-0.73)	<.001	0.80 (0.66-0.98)	0.03	0.71 (0.59-0.86)	<.001
Latinx/Latino/Latina_Hispanic	0.66 (0.55-0.79)	<.001	1.00 (0.82-1.22)	0.99	0.97 (0.81-1.17)	0.76
Middle Eastern or North African	0.54 (0.43-0.69)	<.001	0.83 (0.65-1.06)	0.13	0.82 (0.64-1.03)	0.09
Prefer not to answer	1.17 (1.03-1.33)	0.02	1.34 (1.17-1.53)	<.001	1.36 (1.19-1.55)	<.001
More than 1 race	0.95 (0.79-1.15)	0.62	0.88 (0.72-1.08)	0.22	0.95 (0.78-1.15)	0.57
Of some other race	0.48 (0.34-0.68)	<.001	0.82 (0.57-1.19)	0.30	0.71 (0.50-1.02)	0.06
**Years in Practice Post Training (vs. 1-5 years)**						
6-10 years	1.35 (1.22-1.50)	<.001	1.13 (1.01-1.26)	0.03	1.00 (0.90-1.11)	0.99
11-15 years	1.38 (1.24-1.53)	<.001	1.07 (0.95-1.20)	0.26	0.91 (0.82-1.02)	0.10
16-20 years	1.25 (1.11-1.40)	<.001	0.93 (0.82-1.05)	0.23	0.82 (0.73-0.92)	<.001
20+ years	0.85 (0.77-0.93)	<.001	1.52 (1.38-1.67)	<.001	1.31 (1.19-1.44)	<.001
**Specialty (vs. Primary Care)**						
Hospital Based	0.92 (0.84-1.00)	0.04	1.17 (1.07-1.28)	<.001	1.21 (1.11-1.32)	<.001
Medical Specialty	0.87 (0.79-0.96)	0.003	0.92 (0.83-1.01)	0.08	1.03 (0.94-1.14)	0.53
Surgery Specialty	0.92 (0.83-1.02)	0.12	0.88 (0.79-0.98)	0.02	1.27 (1.14-1.41)	<.001
Obstetrics And Gynecology	0.91 (0.79-1.04)	0.18	0.90 (0.77-1.05)	0.17	0.97 (0.84-1.12)	0.66
Psychiatry	0.63 (0.51-0.78)	<.001	0.95 (0.76-1.19)	0.65	1.31 (1.06-1.61)	0.01

† Pooled due to small sample sizes.

© 2026 Carlasare LE et al. *JAMA Network Open*.
