# Supplementary material for: Sense of Belonging, Burnout, and Work Intentions Among US Physicians
Source: JAMA Netw Open. 2026 Mar 30;9(3):e264171. doi: 10.1001/jamanetworkopen.2026.4171 (PMC13036579; doi:10.1001/jamanetworkopen.2026.4171)
Supplement: Supplement 2. — Data Sharing Statement [file jamanetwopen-e264171-s002.pdf]

Data Sharing Statement

Carlasare. Sense of Belonging, Burnout, and Work Intentions Among US Physicians. *JAMA Netw Open*. Published March 30, 2026. doi:10.1001/jamanetworkopen.2026.4171

Data
Data available: No
